# MRI Guiding of the Middle Cerebral Artery Occlusion in Rats Aimed to Improve Stroke Modeling

**DOI:** 10.1007/s12975-017-0590-y

**Published:** 2017-11-25

**Authors:** Ilya L. Gubskiy, Daria D. Namestnikova, Elvira A. Cherkashova, Vladimir P. Chekhonin, Vladimir P. Baklaushev, Leonid V. Gubsky, Konstantin N. Yarygin

**Affiliations:** 1 Research Institute of Cerebrovascular Pathology and Stroke, Pirogov Russian National Research Medical University, Moscow, Russia; 2 Department of Neurology, Neurosurgery and Medical Genetics, Pirogov Russian National Research Medical University, Moscow, Russia; 3 Serbsky Federal Medical Research Centre of Psychiatry and Narcology, Pirogov Russian National Research Medical University, Moscow, Russia; 4 Federal Research Clinical Center of Specialized Medical Care and Medical Technologies of the FMBA of Russia, Moscow, Russia; 5 Institute of Biomedical Chemistry, Moscow, Russia

**Keywords:** Stroke, Animal model, Magnetic resonance imaging, Endovascular surgery, MCAO

## Abstract

The middle cerebral artery occlusion (MCAO) model in rats closely imitates ischemic stroke and is widely used. Existing instrumental methods provide a certain level of MCAO guidance, but monitoring of the MCA-occluding intraluminal filament position and possible complications can be improved. The goal of this study was to develop a MRI-based method of simultaneous control of the filament position, blood flow in the intracranial vessels, and hemorrhagic complications. Rats were subjected to either MRI-guided MCAO (group 1, *n* = 51) or MCAO without MRI control (group 2, *n* = 38). After operation, group 1 rats were transferred into a MRI scanner for the control of the filament position and possible complications. Ninety minutes after the onset of MCAO, the filament was removed in rats of both groups and MRI control of the infarct volume and hemorrhagic complications performed. High-resolution T1- and T2-weighted imaging performed immediately after filament insertion provided visualization of the filament position, blood flow in brain arteries, and complications related to inappropriate filament insertion. It permitted replacement of wrongly positioned filaments and exclusion of animals with complications from the experiment. MRI-based MCAO guiding provided real-time intra-operational monitoring of crucial parameters determining MCAO suitability for stroke modeling, including better assessment of the operation outcomes in individual animals and significant enhancement of the model success rate. The possibility of simultaneous visualization of the filament, blood flow in the arteries, brain tissue, and hemorrhagic complications is the principal advantage of the proposed method over other instrumental methods of MCAO quality control.

**Graphical Abstract:**
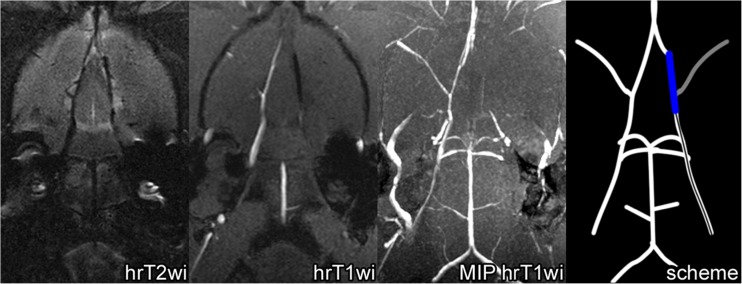
MRI-guided middle cerebral artery occlusion technique permits intra-operational monitoring via direct non-invasive simultaneous visualization of the filament, blood flow in the arteries, brain tissue, and hemorrhagic complications. It provides better assessment of MCAO outcomes in individual animals and significant enhancement of MCAO success rate.

MRI-guided middle cerebral artery occlusion technique permits intra-operational monitoring via direct non-invasive simultaneous visualization of the filament, blood flow in the arteries, brain tissue, and hemorrhagic complications. It provides better assessment of MCAO outcomes in individual animals and significant enhancement of MCAO success rate.

## Introduction

Animal models of ischemic stroke are crucial for understanding complex cellular and molecular mechanisms of the pathogenesis of this debilitating disease and for the design of new approaches for stroke therapy. Among many models developed in a variety of species [1], the intraluminal reversible middle cerebral artery occlusion (MCAO) model in rats is one of the most closely imitating human ischemic stroke [2] and, therefore, frequently used. Actually, in humans, the middle cerebral artery (MCA) and its branches are the most often affected by stroke cerebral vessels (up to 70% of all cerebral infarctions [3]). Moreover, major rat cerebral arteries closely resemble their human counterparts with regard to the structure of the vascular wall and morphological changes associated with cerebral vascular diseases [4]. The operation is minimally invasive and causes relatively low and controllable damage to brain structures [5].

MCAO was first introduced by Koizumi et al. [6] in 1986 and modified by Longa et al. [7] in 1989. Since that time, many further adjustments in surgical techniques [8], anesthesia [9], filament types [10, 11], depth of the MCA-occluding tool (filament) insertion [12, 13], occlusion time [14], and rat strains [15] have been proposed. Despite the improvements, the filament selection and its correct intravascular placement is still a challenge. Inadequate size and position of the filament lead to insufficient MCA occlusion and/or to rupture of intracranial vessels and hemorrhagic complications [16]. Currently, the advancement of the surgical procedure and its results are usually monitored by laser Doppler flowmetry (LDF) [17], less commonly by laser speckle contrast imaging (LSCI) [18], magnetic resonance angiography [16], digital subtraction angiography (DSA) [19, 20], magnetic resonance perfusion [21], computed tomography perfusion [22], and rarely by radionuclide methods [23]. All those methods, with the exception of DSA, estimate blood flow in the cerebral vessels or brain perfusion, but do not provide direct visualization of the filament. DSA requires the use of a contrast agent and ionizing radiation and allows visualization of the filament and blood flow estimate, but does not deliver images of the surrounding brain tissue.

This study describes a method of MRI-guided MCAO, providing direct non-invasive detection and control of the intraluminal position of the filament with simultaneous imaging of brain tissue, surgical complications, and blood flow in the cerebral vessels. This approach facilitates the selection of the filament size and insertion length and increases MCAO success rate.

## Materials and Methods

### Animals

Adult male Wistar rats (*n* = 89) weighing 250–300 g were used for the experiment. Males were chosen to avoid the potential neuroprotective action of estrogens affecting the intensity of stroke manifestations [24]. Animals were obtained from AlCondi, Ltd., Moscow, Russia, and housed in groups of four to five animals per cage before surgery. Rats were maintained under a 12-h/12-h light/dark cycle and had unlimited access to standard rodent chow and water. All manipulations with experimental animals were approved by the local Ethical Committee of the Pirogov Russian National Research Medical University (Protocol No. 140 from December 15, 2014) and were carried out in accordance with the directive 2010/63/EU of the European Parliament and the Council of European Union on the protection of animals used for scientific purposes issued on September 22, 2010. Rats were randomly attributed to one of two experimental groups: (1) MRI-guided MCAO (*n* = 51) and (2) MCAO without control of the filament position (*n* = 38). At the end of the experiment, animals were sacrificed by intraperitoneal injection of a lethal dose of chloral hydrate.

### Middle Cerebral Artery Occlusion Model

**Fig. 1:**
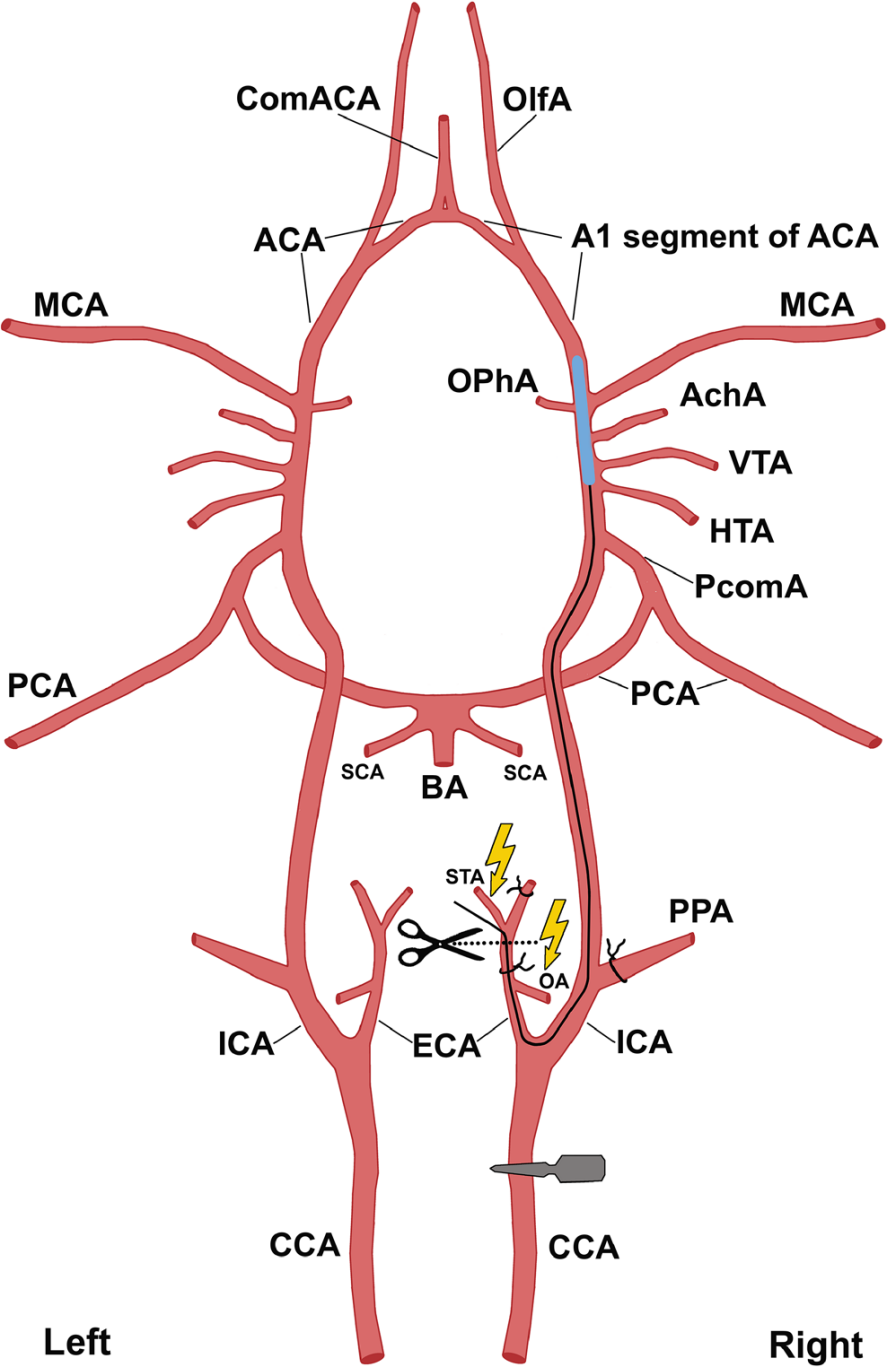
Schematic representation of the major rat head and neck arteries (view from the top) and the appropriate filament position during MCAO. The filament is inserted into ICA through the stump of ECA and pushed forward to occlude the origin of MCA, VTA, and AchA with its silicon-coated tip (blue). OA and ST are electrocoagulated, while PPA, the proximal part of ECA, and the ECA stump ligated. A microsurgical clip is placed on CCA. *ACA* anterior cerebral artery, *AchA* anterior choroidal artery, *BA* basilar artery, *CCA* common carotid artery, *ComACA* common (azygos) anterior cerebral artery, *ECA* external carotid artery, *HTA* hypothalamic artery, *ICA* internal carotid artery, *MCA* middle cerebral artery, *OA* occipital artery, *OlfA* olfactory artery, *OphA* ophthalmic artery, *PCA* posterior cerebral artery, *PcomA* posterior communicative artery, *PPA* pterygopalatine artery, *SCA* superior cerebellar artery, *STA* superior thyroid artery, *VTA* ventral thalamic artery

Rats were anesthetized with 3.5–4% isoflurane and maintained at artificial ventilation with the mixture of 2–2.5% isoflurane and 97.5–98% atmospheric air supplied by the EZ-7000 Classic System animal anesthesia system (E-Z Anesthesia® Systems). Throughout all surgical procedures, body temperature was maintained at 37 °C with a heating pad. Transient right middle cerebral artery occlusion (MCAO) for 90 min was performed using silicon rubber-coated 4-0 monofilament (Doccol Corporation). Major vessels of rat neck and head with introduced filament and a simplified operation scheme are shown in Fig. 1. Each rat was placed in the supine position and a 10-ml syringe was put under the neck for better access to the carotid arteries. The neck was shaved and cleaned with betadine and 70% ethanol. Atropine sulfate 0.05 mg/kg in 1 ml 0.9% NaCl was injected intraperitoneally to reduce respiratory tract secretion and to block the vagus nerve. An injection of 0.1 ml of 0.5% bupivacaine was made at the prospective incision site (ventral neck midline). Dexpanthenol gel (Corneregel®, Dr. Gerhard Mann Chem.-Pharm., Germany) was applied to both eyes to avoid drying. Surgery was performed with microsurgical instruments (Titan surgical, Kazan, Russian Federation).

#### Surgery

1. Ventral midline incision (~ 1 cm) was performed. Superficial fascia was dissected, and the right submandibular glandular tissue was carefully forced aside.
2. Within the center of the triangle formed by the sternomastoid, sternohyoid, and digastric muscles, a blunt dissection was performed to expose the bifurcation of the right common carotid artery (CCA). A retractor was applied to open the surgical field.
3. Under an operating microscope, the vagus nerve and small nerve fibers surrounding the arteries were carefully anesthetized with a cotton bud impregnated with 0.1–0.2 ml of 0.5% bupivacaine for additional analgesia and prevention of the acute respiratory distress syndrome. The vagus nerve was bluntly separated from the CCA and the internal carotid artery (ICA). During all subsequent procedures, mechanical contact with the vagus nerve was minimized.
4. The ССA, ICA with the pterygopalatine artery (PPA), and the external carotid artery (ECA) with its two branches (superior thyroid artery (STA) and occipital artery (OA)) were separated bluntly from the surrounding fascia, adipose tissue, and small nerves.
5. A microsurgical clip was placed on the CCA at 0.5 cm from the bifurcation. Thread ligatures were avoided in order to prevent mechanical trauma of the intima and thrombosis. STA and OA were cauterized to avoid bleeding after cutting the ECA and for better surgical access to the bifurcation.
6. The PPA was ligated with a 5-0 silk suture and two 5-0 silk sutures were placed around the ECA: a tight ligature was put as distally as possible from the bifurcation and one more, loose ligature—0.2–0.3 mm distally from the bifurcation. The second microsurgical clip was temporarily placed on the ICA to prevent retrograde bleeding after cutting the ECA.
7. The ECA was then cut with microscissors between two sutures and a silicon rubber-coated size 4-0 monofilament (Doccol Corporation, diameter 0.19 mm, length 30 mm; diameter with coating 0.37 ± 0.02 mm; coating length 3–4 mm) was inserted into the stump of the ECA and guided towards ICA where the microsurgical clip was located. Then, the silk suture around the ECA stump was tightened in order to prevent bleeding, microclip from the ICA was removed, and monofilament was advanced for 18–20 mm into the lumen of ICA towards the middle cerebral artery (MCA) until mild resistance was felt. MCAO started at this moment and continued for 90 min. The major vessels of the neck and head of the rat with introduced filament and the operation scheme are shown in Fig. 1. The elongated rubber-coated monofilament tip was chosen to avoid adhesion to the blood vessel wall and to ensure MCA occlusion regardless the anatomical variations of the site and form of its origin [5, 25].
8. The microsurgical clip was removed from the CCA. The removal of microsurgical clips before MRI was necessary to avoid magnetic susceptibility artifacts. The incision was closed with a simple interrupted 3-0 silk suture.
9. After the wound closure, rats from the experimental group 2 were placed in a preheated cage for recovery from anesthesia, and at the end of the occlusion period, the filament was removed (see point 10). Each rat from the experimental group 1 was transferred into the MRI scanner for control of the filament position and for revealing the hemorrhagic complications. There were three possible outcomes:
  A In case of successful filament placement with complete occlusion of the right MCA confirmed by MRI, the rat was placed in a preheated cage (with the heating pad under it) for recovery from anesthesia.
  B In case of incorrect filament position with persistent blood flow in the right MCA, the rat was transferred back to the surgical workplace for the replacement of the filament (two attempts maximum). After disinfection, the incision was reopened, and a microsurgical clip was again placed on the CCA. The filament was slowly advanced into the lumen of ICA or withdrawn from it depending on the MRI results. Further procedures were repeated from the step 8.
  C In case of hemorrhagic complications (subdural or subarachnoid hemorrhage), the rat was immediately excluded from the experiment by the intraperitoneal injection of a lethal dose of chloral hydrate.
10. Ten minutes before the end of the occlusion period, all rats from both experimental groups were re-anesthetized. The incision site was again disinfected with betadine and 70% ethanol and the incision was reopened.
11. A microsurgical clip was once more placed on the CCA. The monofilament was slowly withdrawn from ICA until its coated white end was visible through the ICA. At that moment, a microsurgical clip was placed on the ICA distally to the filament tip, and the filament was then completely removed from the ECA stump. The suture on the ECA stump was tightly tied.
12. Both microsurgical clips were removed. The operating wound was rinsed with sterile saline and closed with 3-0 silk suture using a simple interrupted pattern.
13. To provide rehydration, 3 ml of sterile saline was injected intraperitoneally. Each rat was placed in a single cage for recovery from anesthesia. A heating pad was placed under the cage for 30 min.

### MRI

**Fig. 2:**
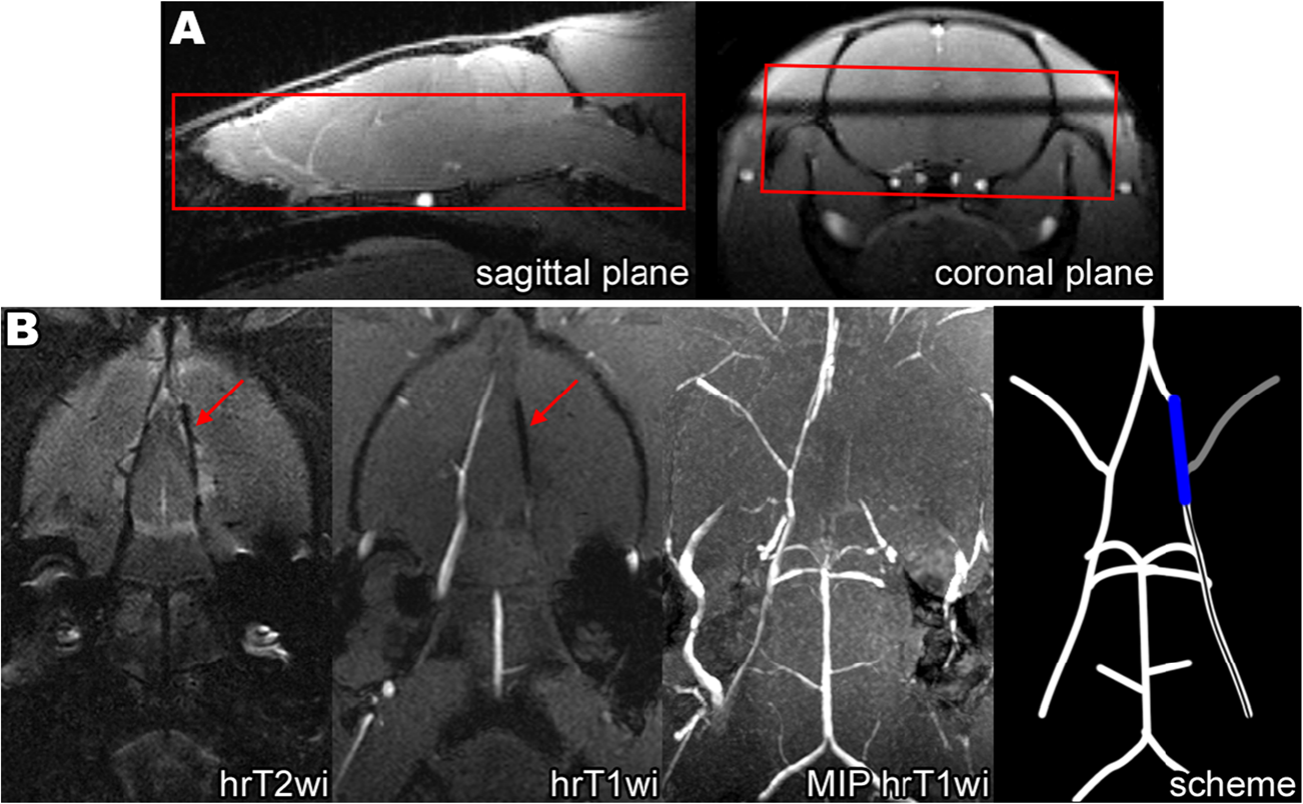
**a** Slab positioning (limited by the red lines) of high-resolution T2wi and high-resolution T1wi of the rat brain performed immediately after MCAO. Slicing was performed in the coronal plane parallel to the intracranial segment of ICA. The skull base served as the inferior slab border. **b** MRI control of the filament position during MCAO: an example of proper filament insertion. Left to right: hrT2wi—high-resolution T2-weighted image, hrT1wi—high-resolution T1-weighted image, MIP hrT1wi—MIP (maximum intense projection) of high-resolution T1-weighted images, and scheme—scheme of the intra-arterial filament position. The silicone-coated filament tip could be visualized as a hypointense intraluminal object (hrT1wi, hrT2wi, blue on scheme) in the distal part of the right ICA and proximal part of the A1 segment of the right ACA. MIP hrT1wi clearly shows the absence of blood flow in the right MCA (second from the right panel, gray on scheme). Red arrows indicate the filament position

MCAO surgery was guided by MRI conducted with the 7-T ClinScan system for small animals (Bruker BioSpin, USA). Each group 1 rat (*n* = 51) was placed in the MR scanner immediately after filament insertion, and high-resolution T2-weighted images (hrT2wi)—Turbo Spin Echo, TR\TE = 3830\54 ms, voxel size 0.1 × 0.1 × 0.3 mm, turbo factor = 12, and high-resolution T1-weighted images (hrT1wi) with reconstruction of maximum intensity projection (MIP)—Gradient Echo, TR\TE = 16\3.1 ms, voxel size 0.125 × 0.129 × 0.2 mm in coronal plane (parallel to the intracranial segment of ICA) were obtained. An example of the slab position is given in Fig. 2a.

The second MRI examination was performed 24 h after MCAO for all animals of both experimental groups (*n* = 48 for group 1 and *n* = 38 for group 2), except 3 rats in group 1 with hemorrhagic complications revealed during the first, intra-operative MRI. The following sequences were obtained: DWI with ADC (diffusion-weighted image with calculation of apparent diffusion coefficient maps TR/TE = 9000/33 ms, b factors = 0 and 1000 s/mm^2^, voxel size 0.35 × 0.35 × 1.0 mm), T2-weighted image (Turbo Spin Echo, turbo factor = 10, TR/TE = 5230/46 ms, voxel size 0.117 × 0.13 × 0.7 mm), and SWI (susceptibility-weighted imaging, 3D Gradient Echo with RF spoiling and flow compensation, TR/TE = 33/17 ms, flip angle = 15, voxel size 0.117 × 0.117 × 0.5 mm) or T2*wi (Gradient Echo-Planar Imaging, TR/TE = 4000/30 ms, flip angle = 60, voxel size 0.22 × 0.22 × 1 mm).

### MRI Data Analysis

MRI morphometry was performed using ImageJ software (Rasband, W.S., ImageJ, U. S. National Institutes of Health, Bethesda, Maryland, USA, http://imagej.nih.gov/ij/, 1997–2015). The volume (*V*) of the infarct zone was calculated utilizing the T2-weighted images taken 24 h after MCAO by the summation of volumes measured in adjacent cross sections according to the following formula: *V* = (S1 + … + S*n*) × (*h* + *d*), where S1,...,S*n* is area measured on slice *n*, *h* is the slice thickness, and *d* is the interval between the slices.

### Statistical Analysis

Comparisons between groups were performed by two-sided Fisher’s exact test. *P* value < 0.05 was considered significant. Experimental groups were originally designed to be equal (38 vs 38 rats) according to the sample size calculation for two-sided Fisher’s exact test using MedCalc Software bvba (type I error = 0.05, type II error = 0.20). However, after detecting an interesting and unexpected phenomenon—partial blood flow in the distal part of the MCA—in some rats, the number of animals in the group 1 has been increased to study it more accurately.

## Results

In our study, the MRI control of MCAO was performed immediately after filament insertion and closure of the surgical wound. Three major operation outcomes were observed: the correct filament position with complete occlusion of the origin of MCA, excessively deep filament insertion resulting in the occlusion of ACA and hemorrhagic complications, and incomplete or excessive filament insertion with continuing blood flow in the MCA.

In case of correct filament insertion, its coated tip stops between the distal part of ICA and the A1 segment of ACA. In this position, it completely blocks direct blood flow from ICA to MCA and the reverse blood flow from ACA. An example of properly inserted filament visualized by MRI is shown in Fig. 2b. The inserted filament and its tip look hypointense on hrT1wi and hrT2wi (Fig. 2b). Importantly, comparison of hrT1wi and hrT2wi MR images allows simultaneous determination of the filament location and detection of blood flow in cerebral blood vessels. On hrT2wi, the cerebrospinal fluid (CSF) filling the subarachnoid spaces is normally visualized as hyperintense areas around the hypointense vessels (Fig. 2b, hrT2wi). If there is no blood flow in the vessels, they are hypointense on both hrT2wi and hrT1wi; if the blood flow is maintained, the vessels are hypointense on hrT2wi and hyperintense hrT1wi. It is illustrated in Fig. 2b, where the occluded right MCA and distal part of the ICA are hypointense on hrT2wi and hrT1wi, while the left MCA with maintained blood flow is hypointense on hrT2wi, but hyperintense on hrT1wi.

**Fig. 3:**
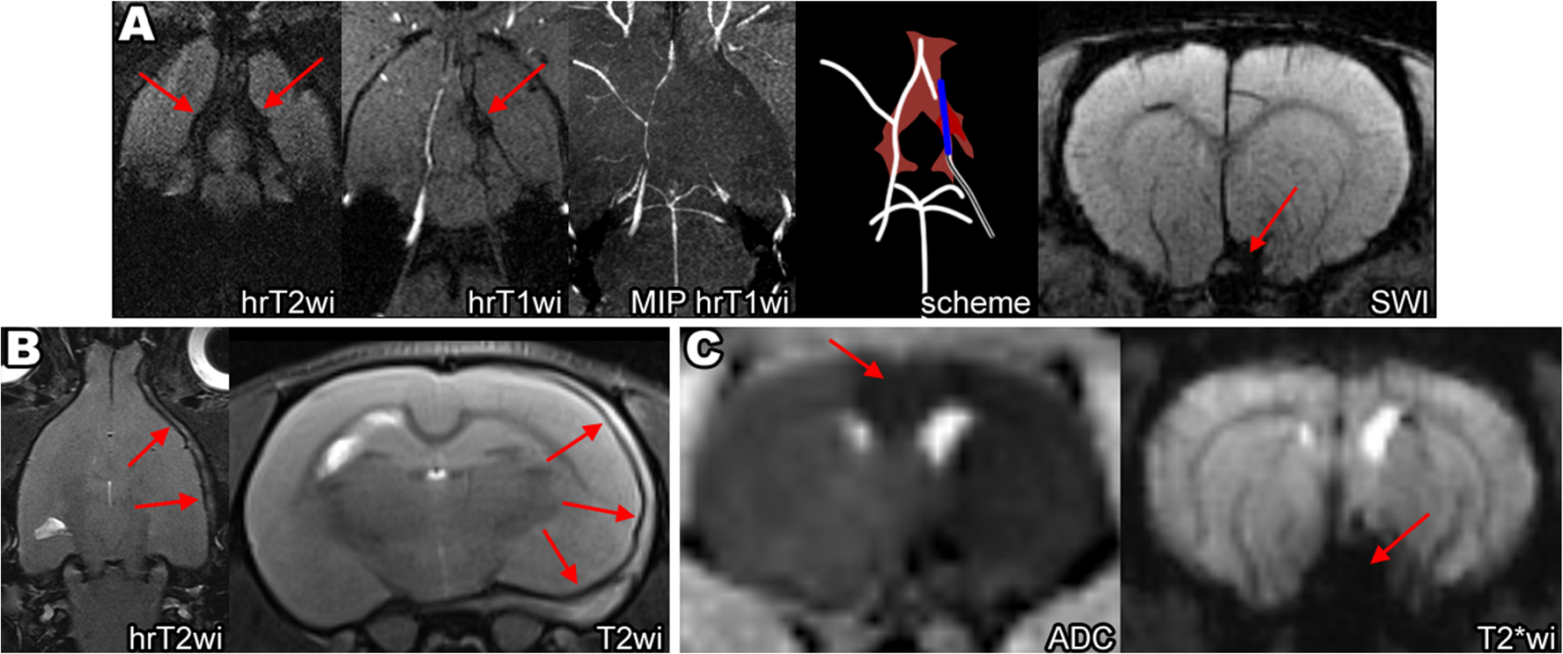
Hemorrhagic complications of MCAO after excessively deep filament insertion. **a** Subarachnoid hemorrhage (SAH). On T2wi, the normal hyperintense signal from the cerebrospinal fluid (CSF) filling the subarachnoid space and cisterns turns into the hypointense signal due to the replacement of CSF with blood (red arrows on hrT2wi, red on scheme). SAH also can be visualized on SWI (red arrow on SWI) and, partly, on hrT1wi (red arrow in hrT1wi). The most common cause of SAH during MCAO is the rapture of the A1 segment of ACA by the filament tip, which in turn causes vasospasm—on MIP hrT1wi right MCA, and the distal part of ICA is not visualized. **b** Subdural hemorrhage (SDH). Subdural hemorrhage, often leading to brain dislocation, can be visualized as a heterogeneous crescent-shaped mass between the dura mater and arachnoid mater (red arrows on hrT2wi and T2wi). **c** Ischemic stroke after rupture of the left ACA by the filament tip. ADC is low not only in the MCA but also in the ACA (red arrow on ADC) vascular territories. SAH visualized on T2*wi as hypointense area in the skull base (red arrow on T2*wi)

Excessive insertion of the filament tip can cause the rupture of intracranial vessels and hemorrhagic complications. The A1 segment of ACA is the predominant injury location, because it is thinner than the distal part of ICA, it bends to merge with the opposite ACA to form common (azygos) anterior cerebral artery, and the filament tip is too thick to fit its inner diameter. MRI images of intracranial hemorrhagic complications after excessive filament insertion are shown on Fig. 3a, b. In our experiments, subarachnoid hemorrhages (SAH) were the most frequent of the observed complications. Normally, the subarachnoid space between the arachnoid membrane and the pia mater contains CSF, which is hyperintense on hrT2wi. In case of SAH, the subarachnoid space becomes replete with blood which is hypointense on the T2wi and SWI as illustrated by Fig. 3a. A particularly deep insertion of the filament can cause subdural hemorrhage appearing as a crescent-shaped mass consisting of liquid and clotted blood between the dura mater and arachnoid mater and leading to brain dislocation (Fig. 3b). Rupture of cerebral blood vessels causes severe vasospasm (Fig. 3a, MIP hrT1wi), which in turn may trigger ischemic stroke (usually in the MCA and the ACA vascular territories as shown in Fig. 3c) mimicking successful MCAO. In our experiments, animals with intracerebral bleeds combined with ischemic stroke exhibited more severe neurological symptoms (including seizures, meningeal signs, impaired consciousness) than rats after correctly performed MCAO (unpublished results). Animals with hemorrhagic complications were excluded from the study immediately after MRI.

**Fig. 4:**
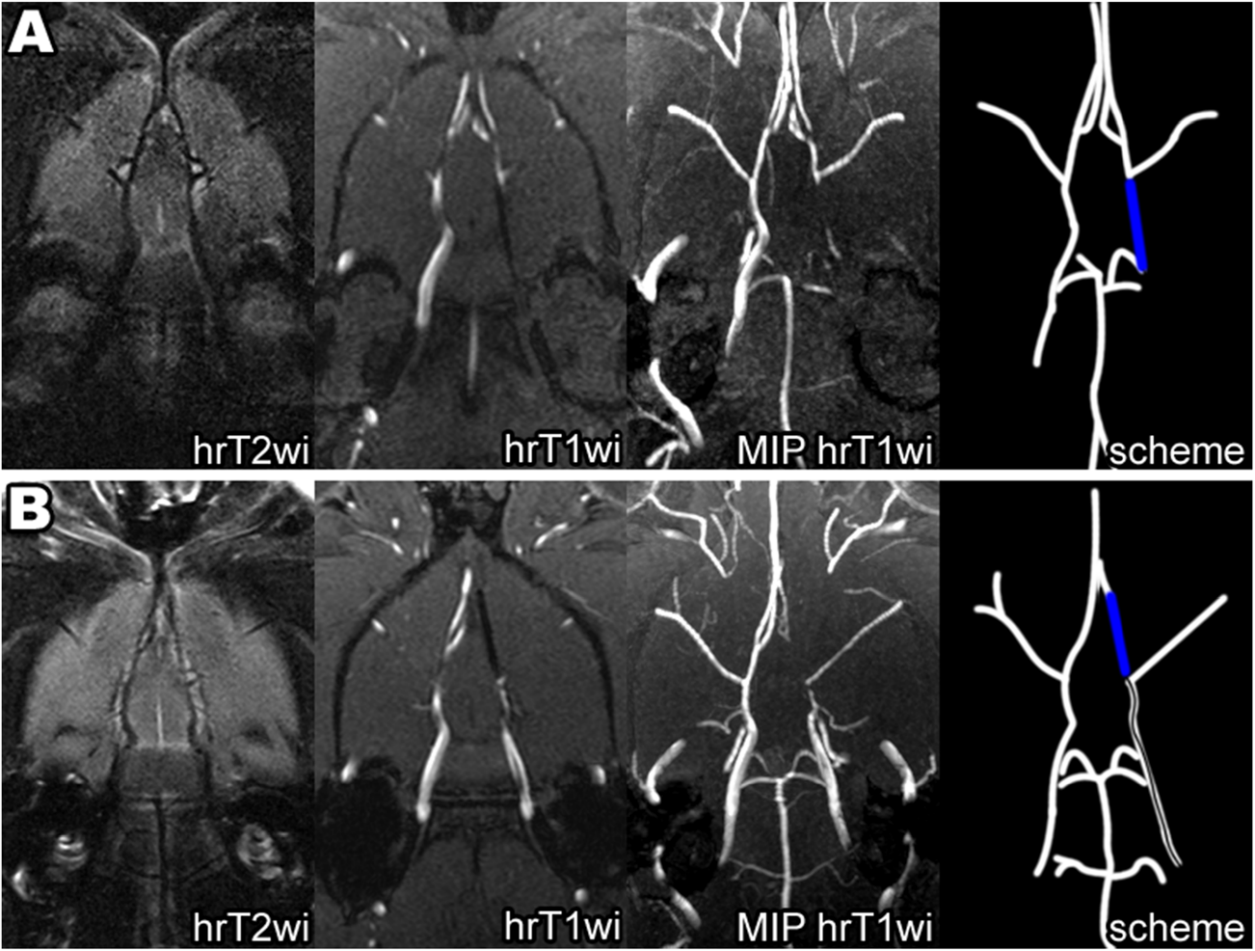
Filament visualization on MRI during MCAO: the examples of incorrect filament position with persistent blood flow in the right MCA. **a** The filament tip inserted insufficiently deep and visualized as elongated hypointense zone inside the distal part of ICA below the origin of MCA on hrT1wi and as the absence of blood flow in the distal part of the ICA on MIP hrT1wi (blue line on scheme). The right MCA is hypertensive on hrT1wi due to the of retrograde blood flow from ACA. **b** Excessively deep filament insertion into ACA is visualized as elongated hypointense zone inside the A1 segment of ACA on hrT1wi and as the absence of blood flow in the A1 segment of ACA on MIP hrT1wi (blue line on scheme). The right MCA is also hypertensive on hrT1wi due to direct blood supply from the ICA

In case of insufficiently deep insertion of the filament, its coated tip does not occlude the origin of the MCA from ICA. On T1wi, MCA with the persistence of retrograde blood flow from ACA is hyperintense in contrast with the hypointensive filament tip located in ICA proximally (Fig. 4a). The reverse situation is possible, when the filament tip goes too far into the ACA (but does not rapture it) and the blood flow in the MCA is preserved. Such filament position is visualized as hyperintense MCA on T1wi and hypointense filament tip in the A1 segment of ACA (Fig. 4b). In all cases of the incorrect filament position without hemorrhagic complications, the replacement of the filament can be performed. The distance between the tip of the filament and the origin of the MCA can be measured on T1wi, and the filament can be repositioned based on this measurement. To avoid thrombosis due to mechanical trauma of arterial intima, we made no more than two filament positioning attempts (1 attempt in 45 rats and 2 attempts in 3 rats).

**Fig. 5:**
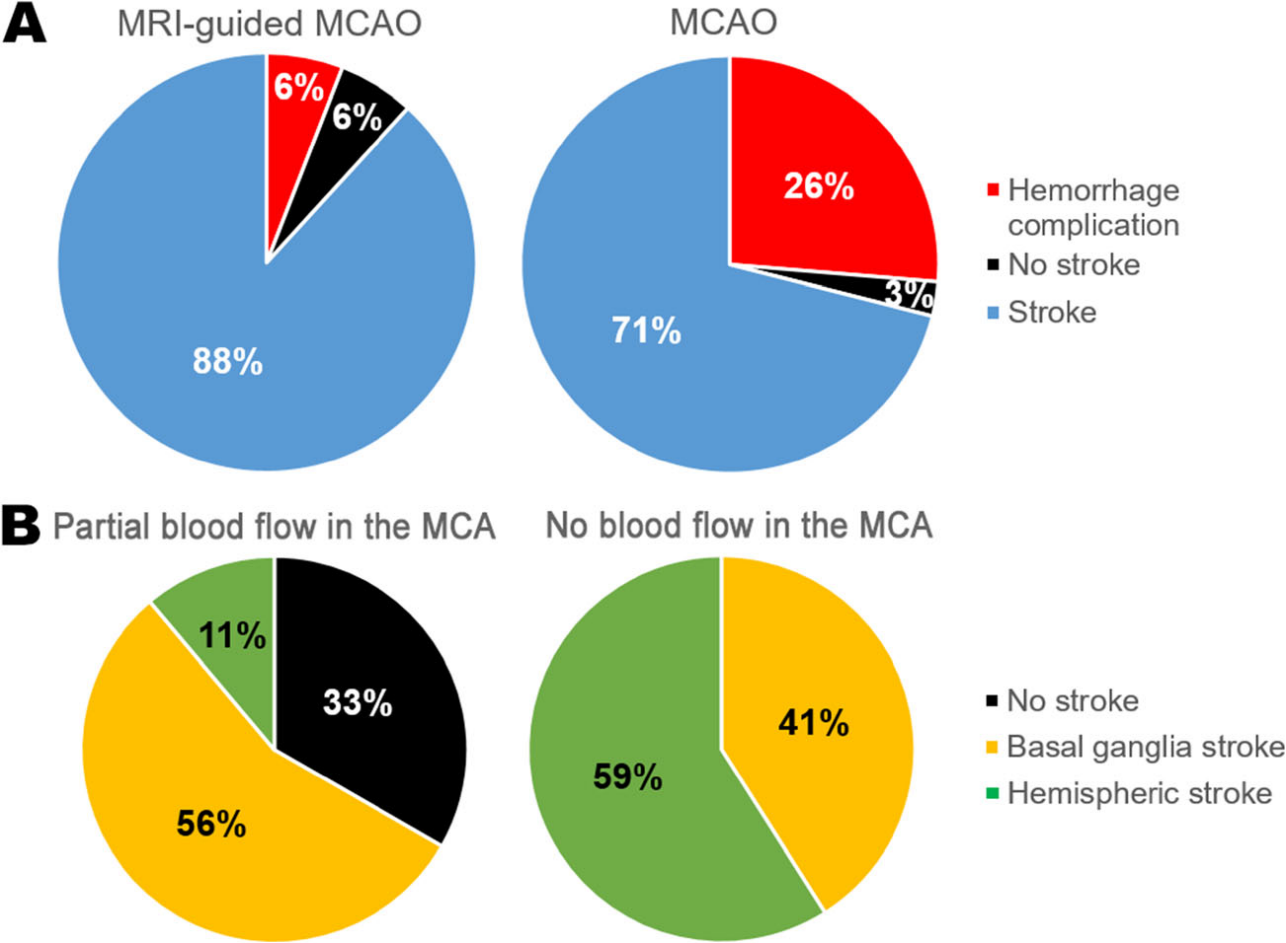
Outcomes of MCAO. **a** Diagrams demonstrate percentages of the successful stroke formation, hemorrhagic complications, and no stroke (or very small ischemic lesion) in group 1 with MRI-guided MCAO and group 2 without the control of the filament position 24 h after MCAO. **b** Outcomes of MCAO depending on the presence or absence of the partial blood flow in the distal part of the MCA. Diagrams demonstrate distribution of rats according to the infarct zone size 24 h after MCAO in group with partial blood flow in the MCA (*n* = 9) and without (*n* = 39)

MCAO was performed in 89 animals: in 51 rats from group 1 with MRI-guided MCAO and in 38 rats from group 2 without control of the filament position. In group 1, hemorrhagic complications were detected in 3 rats (6%) (2 cases of subarachnoid hemorrhages and 1 case of subdural hematoma), no stroke (or very small ischemic lesion) in 3 rats (6%), and successful stroke formation in 45 rats (88%). In group 2, hemorrhagic complications, no stroke, and successful stroke formation were observed in 10 (26%), 1 (3%), and 27 (71%) rats, respectively (Fig. 5a). The frequency of hemorrhagic complications in group 1 was significantly lower (*p* < 0.05).

**Fig. 6:**
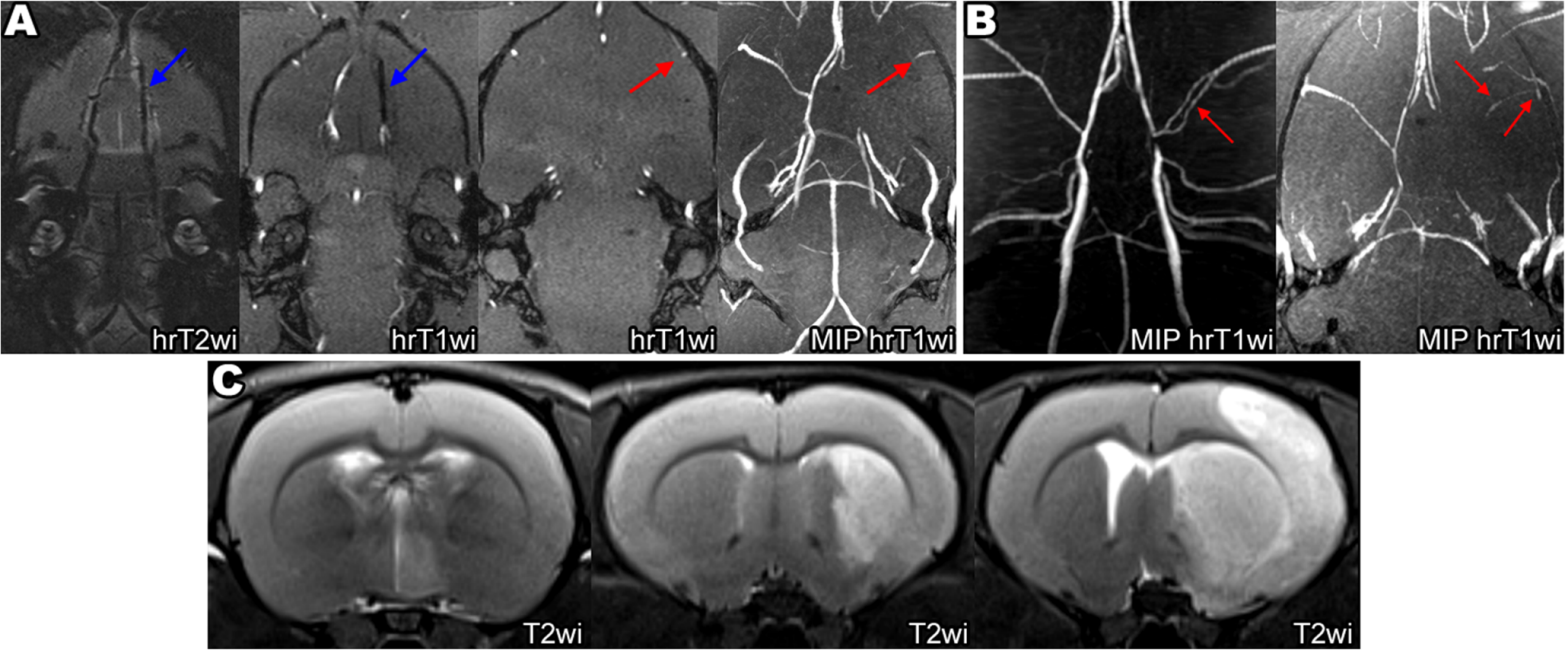
**a** Partial blood flow in the MCA despite the correct position of the filament. The filament tip located correctly with overlapping the origin of the right MCA (blue arrows on hrT2wi and left hrT1wi) and with the absences of blood flow in the proximal part of the artery. Nevertheless, hyperintense signal from the blood flow in the distal part of the right MCA (red arrows on right hrT1wi and MIP hrT1wi) can be observed. **b** Some reasons of persistence of the partial blood flow. MCA fenestration, red arrow on left image. Opening of leptomeningeal anastomosis, red arrow on right image. **c** The examples of different stroke sizes 24 h after MCAO on T2wi. Left to right: 1st image—small hyperintense on T2WI ischemic lesion in hypothalamic area is visualized, 2nd image—basal ganglia stroke, and 3rd image—hemisphere stroke

At the first MRI examination in some animals from group 1, partial blood flow in the distal part of MCA was observed despite the correct position of the filament with complete occlusion of the origin of MCA (Fig. 6a). Depending on the presence or absence of this phenomenon, all rats were attributed to one of two groups and distribution of animals according to the infarct zone size 24 h after MCAO in both groups was estimated (Fig. 5b). In the group with preserved partial MCA blood flow (*n* = 9), the distribution was as follows: 3 rats (33%) had no stroke, 5 rats (56%) had basal ganglia stroke, and 1 rat (11%) had hemispheric stroke. In the group without MCA blood flow, all animals (*n* = 39) developed ischemic stroke: 16 rats (41%) in basal ganglia region only and 23 rats (59%) with the involvement of the whole MCA blood supply territory (hemispheric stroke). The frequency of hemispheric stroke was significantly higher in the second group (*p* < 0.05). The reason for the maintenance of partial blood flow could be the anomalous vascular architecture [10], including MCA fenestration (red arrow on Fig. 6b, left) or opening of leptomeningeal anastomosis (red arrow on Fig. 6b, right). The examples of three different stroke volumes 24 h after MCAO are given in Fig. 6c. The mean volume of cerebral infarction 24 h after MCAO in the group without partial blood flow in the MCA was 49 ± 15 mm^3^ in rats with basal ganglia infarction and 197 ± 86 mm^3^ in rats with hemispheric stroke.

## Discussion

Application of instrumental methods for intra-operative monitoring of crucial parameters can increase the success rate of MCAO stroke modeling. Currently, laser Doppler flowmetry (LDF) is the most widely used approach [26]. LDF allows dynamic control of brain perfusion, including regional cerebral blood flow (rCBF) in the MCA vascular territory, and is quite helpful. Still, more accurate measurement of rCBF with higher spatial and temporal resolution can be performed using laser speckle contrast imaging (LSCI) [18]. However, LDF and LSCI do not show the filament intraluminal position and cannot directly detect hemorrhagic complications. Moreover, these methods are relatively invasive (LDF requires drilling of the burr hole in the skull, while LSCI—only skull thinning) and animals need fixation and general anesthesia during the entire period of MCA occlusion. Uninterrupted anesthesia can reduce the infarct size due to anesthetic-induced neuroprotection [27]. Unlike LDF and LSCI, the MRI-based approach described in this paper is non-invasive and takes about 10 min, after which anesthesia can be stopped. Though MRI does not deliver direct rCBF estimation, it provides detection of blood flow in the MCA together with direct visualization of the filament position and hemorrhagic complications.

There are other instrumental methods in use allowing rCBF estimation during MCAO modeling, but not filament or hemorrhage visualization. They include contrast CT perfusion [22] and MR perfusion [21] requiring intravenous catheter placement for bolus contrast injection. Arterial spin labeling, a non-invasive contrast-free MRI technique [22, 28], and radionuclide perfusion methods with intravenous administration of radiopharmaceuticals [23] are also used.

Magnetic resonance angiography (MRA) [16] and digital subtraction angiography (DSA) [19, 20] allow the assessment of MCAO success by visualization of blood flow in the MCA. MRA is a non-invasive contrast-free method providing indirect detection of the filament position based on the absence of blood flow in MCA, while DSA is an interventional technique delivering direct filament imaging, but requiring contrast injection for vessel visualization [19, 20]. The disadvantages of DSA include the inability to visualize soft tissue and the operator’s exposure to ionizing radiation. The MRI-based method presented here provides both visualization of blood flow and soft tissue contrast permitting the imaging of the filament and surrounding brain structures. Adequate soft tissue contrast makes possible the early detection of hemorrhagic complications occurring in up to 40% of rats undergoing MCAO [17, 29]. In our experiments, the MRI control significantly reduced the frequency of hemorrhagic complications, thus improving the success rate of stroke modeling.

MRI allows detection of continuing partial blood flow in the distal part of MCA during MCAO, recognition of the resulting reduction of stroke volume and frequency of hemispheric strokes, and exclusion of affected rats from the experiment. Some of the possible reasons of the maintenance of this residual blood flow include variations in the cerebral arteries anatomy (Fig. 6b, left) and opening of the leptomeningeal anastomosis (Fig. 6b, right).

The main disadvantages of the method suggested here include high costs of MRI scanners and the need of close location of MRI scanner and the operating room necessary for conducting the intra-operative imaging. On the other hand, it was reported that the unmonitored MCAO outcomes vary greatly in different rat strains [15, 30, 31] and even in rats of one breed from different suppliers [15]. Therefore, MRI guidance is essential to determine the required filament insertion depth and significantly improve the reproducibility of stroke modeling.

## Conclusions

The proposed MRI-guided MCAO technique permits intra-operational monitoring via direct non-invasive visualization of the filament intraluminal position, blood flow in the intracranial vessels, and hemorrhagic complications, significantly improving the success rate of MCAO stroke modeling. Its inability to directly assess brain perfusion is counterweighed by the capacity to detect blood flow in brain arteries.
